# Supplementary material for: Recommended survey designs for occupancy modelling using motion-activated cameras: insights from empirical wildlife data
Source: PeerJ. 2014 Aug 28;2:e532. doi: 10.7717/peerj.532 (PMC4157302; doi:10.7717/peerj.532)
Supplement: Table S1 — Optimal survey design for estimating occupancy with three levels of acceptable error defined by the root mean squared error (RMSE). In the main analysis (Table 1) we considered the costs of cameras and occasions to be equivalent and determined optimality solely on the minimum number of survey days (sites × occasions). Here we provide a scenario that also considers the financial costs of conducting a motion-activated camera survey, with lower cost survey designs that meet the desired level of precision being selected in preference over more expensive approaches. For the purpose of our scenario, a motion-activated camera = 250, while the cost of surveying an occasion at = 10, which incorporates the cost of personnel, vehicle use, batteries and maintenance of the equipment. See Spreadsheet S1 to calculate alternate scenario costs. [file peerj-02-532-s001.docx]

|  |  |  | **RMSE 0.15** | | | **RMSE 0.10** | | | **RMSE 0.05** | | |
| --- | --- | --- | --- | --- | --- | --- | --- | --- | --- | --- | --- |
| **Species** | Ψ | P | Sites^1^ x occasions | Total Survey effort | Cost  ($) | Sites^1^ x occasions | Total Survey effort | Cost ($) | Sites^1^ x occasions | Total Survey effort | Cost ($) |
| **Spotted Skunk** | 0.245 | 0.023 | 20 x 100 | 2000 | 6000 | 20 x 120 | 2400 | 6200 | 40 x 120 | 4800 | 11200 |
| **Elk** | 0.585 | 0.024 | 20 x 80 | 1600 | 5800 | 30 x 120 | 3600 | 8700 | 60 x 100 | 6000 | 16000 |
| **Mountain Lion** | 0.600 | 0.030 | 20 x 80 | 1600 | 5800 | 30 x 100 | 3000 | 8500 | 50 x 120 | 6000 | 13700 |
| **Coyote** | 0.861 | 0.031 | 10 x 60 | 600 | 3100 | 20 x 80 | 1600 | 5800 | 30 x 80 | 2400 | 8300 |
| **Bobcat** | 0.970 | 0.040 | 10 x 40 | 400 | 2900 | 10 x 60 | 600 | 3100 | 10 x 80 | 800 | 3300 |
| **Gray Fox** | 0.400 | 0.063 | 10 x 80 | 800 | 3300 | 30 x 40 | 1200 | 7900 | 40 x 120 | 4800 | 11200 |
| **Black Bear** | 0.504 | 0.072 | 20 x 40 | 800 | 5400 | 30 x 40 | 1200 | 7900 | 50 x 40 | 2000 | 12900 |
| **Virtual sp. 1** | 0.200 | 0.120 | 10 x 40 | 400 | 2900 | 20 x 40 | 800 | 5400 | 30 x 60 | 1800 | 8100 |
| **Virtual sp. 2** | 0.400 | 0.l60 | 10 x 40 | 400 | 2900 | 30 x 20 | 600 | 7700 | 40 x 80 | 3200 | 10800 |
| **Virtual sp. 3** | 0.600 | 0.120 | 10 x 40 | 400 | 2900 | 30 x 40 | 1200 | 7900 | 40 x 80 | 3200 | 10800 |
| **Mule Deer** | 0.925 | 0.141 | 10 x 20 | 200 | 2700 | 10 x 20 | 200 | 2700 | 20 x 20 | 400 | 5200 |
| **Cottontail Rabbit** | 0.925 | 0.190 | 10 x 20 | 200 | 2700 | 10 x 20 | 200 | 2700 | 20 x 20 | 400 | 5200 |

^1^ Sites are the number of cameras and occasions are the number of survey days at each site.
