# Supplementary material for: Recommended survey designs for occupancy modelling using motion-activated cameras: insights from empirical wildlife data
Source: PeerJ. 2014 Aug 28;2:e532. doi: 10.7717/peerj.532 (PMC4157302; doi:10.7717/peerj.532)
Supplement: Table S2 — The probability (p∗∗) of capturing a photograph of a very rare species (ψ = 0.05 and p = 0.05) as a function of the number of occasions and sites (a), where (p∗∗ = 1−[1−ψ(1−(1−p)S)]N). The survey cost in dollars calculated on the basis that each camera is 250 and a survey occasion costs 10 (b). See Spreadsheet S1 to calculate alternate scenario costs. [file peerj-02-532-s002.docx]

(a)

| **Sites (cameras)** | | | | | | | | | |
| --- | --- | --- | --- | --- | --- | --- | --- | --- | --- |
| **Occasions (days)** | **10** | **20** | **30** | **40** | **50** | **60** | **70** | **80** | **120** |
| **20** | 0.278 | 0.479 | 0.624 | 0.729 | 0.804 | 0.859 | 0.898 | 0.926 | 0.980 |
| **40** | 0.360 | 0.590 | 0.737 | 0.832 | 0.892 | 0.931 | 0.956 | 0.972 | 0.995 |
| **60** | 0.387 | 0.624 | 0.769 | 0.858 | 0.913 | 0.947 | 0.967 | 0.980 | 0.997 |
| **80** | 0.396 | 0.635 | 0.780 | 0.867 | 0.920 | 0.951 | 0.971 | 0.982 | 0.998 |
| **100** | 0.399 | 0.639 | 0.783 | 0.870 | 0.922 | 0.953 | 0.972 | 0.983 | 0.998 |
| **120** | 0.401 | 0.641 | 0.785 | 0.871 | 0.923 | 0.954 | 0.972 | 0.983 | 0.998 |

(b)

| **Sites (cameras)** | | | | | | | | | |
| --- | --- | --- | --- | --- | --- | --- | --- | --- | --- |
| **Occasions (days)** | **10** | **20** | **30** | **40** | **50** | **60** | **70** | **80** | **120** |
| **20** |  |  |  |  |  |  |  |  | $30200 |
| **40** |  |  |  |  |  |  | $17900 | $20400 | $30400 |
| **60** |  |  |  |  |  |  | $18100 | $20600 | $30600 |
| **80** |  |  |  |  |  | $15800 | $18300 | $20800 | $30800 |
| **100** |  |  |  |  |  | $16000 | $18500 | $21000 | $31000 |
| **120** |  |  |  |  |  | $16200 | $18700 | $21200 | $31200 |

Shaded areas indicate scenarios with a probability ≥ 0.95 of capturing a photograph of a very rare species.
