# Supplementary figures and images for: Recommended survey designs for occupancy modelling using motion-activated cameras: insights from empirical wildlife data
Source: PeerJ. 2014 Aug 28;2:e532. doi: 10.7717/peerj.532 (PMC4157302; doi:10.7717/peerj.532)

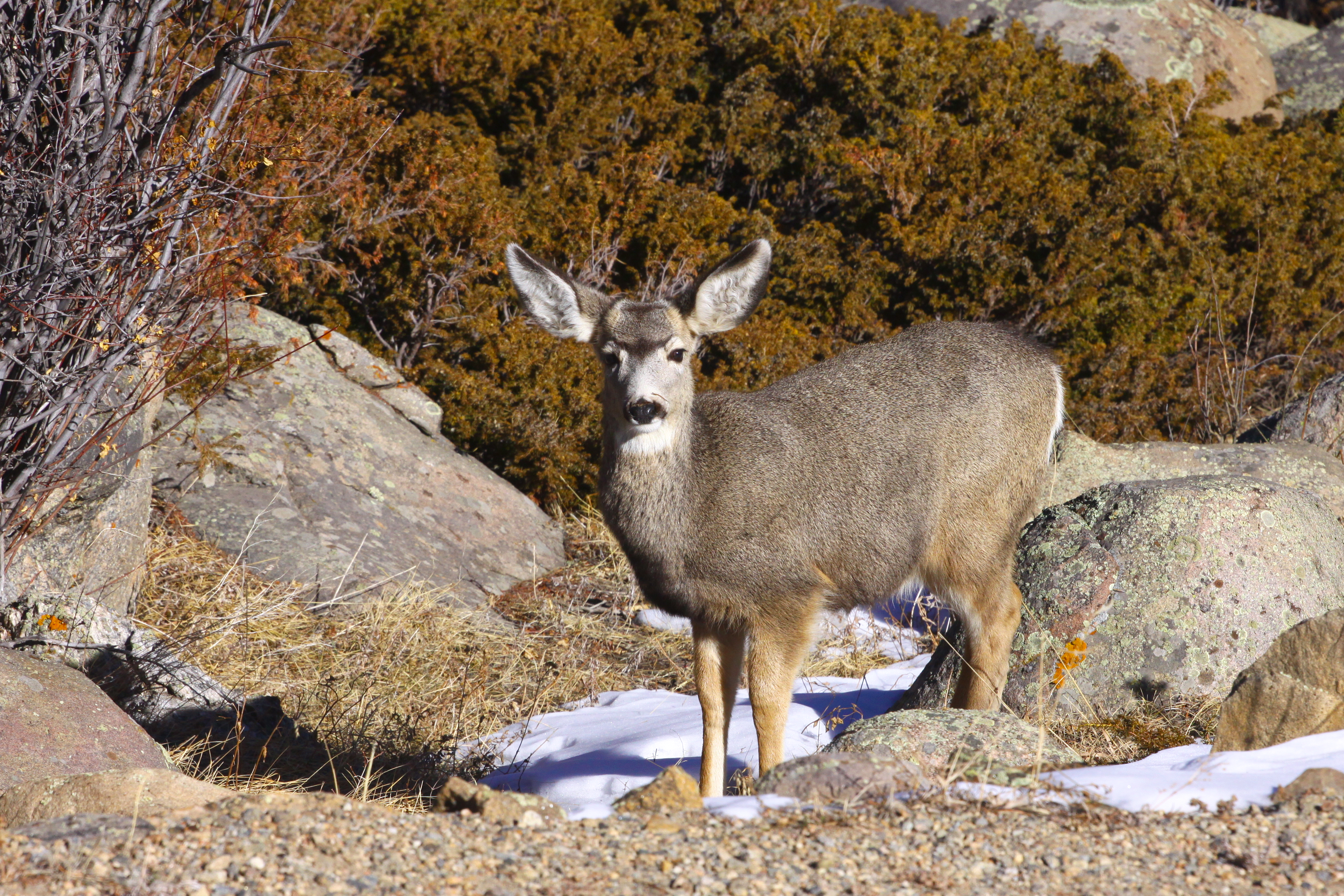

Supplement: Table S4 — For consideration as an image to be used on the peerJ website. Credit: Graeme Shannon. Manuscript 2014:06:2254:1:0:NEW The mule deer is one of the species that we explore in our camera trap study and represents our research. [file peerj-02-532-s004.jpg]
